# An exploration of perceptions and preferences for healthy eating in Dutch consumers: a qualitative pilot study

**DOI:** 10.1186/s40814-020-00735-6

**Published:** 2021-01-08

**Authors:** Juul M. J. Coumans, Catherine A. W. Bolman, Lilian Lechner, Anke Oenema

**Affiliations:** 1 Department of Health Psychology, Faculty of Psychology, Open University of the Netherlands, Heerlen, The Netherlands; 2 Department of Health Promotion, Care and public health research institute (Caphri), Faculty of Health Medicine and Life Sciences, Maastricht University, Maastricht, The Netherlands

**Keywords:** Dietary guidelines, Perceptions on healthy eating, Dutch consumers, Qualitative research

## Abstract

**Background:**

Unhealthy dietary patterns are highly prevalent in Western countries, and they have been associated with depression, hypertension, heart disease, cancer, type 2 diabetes, and obesity. Many dietary interventions have been developed to promote healthier dietary behavior, yet most do not achieve the intended dietary change. This study aims to provide a better understanding of what Dutch consumers perceive as a healthy diet, how this relates to the current Dutch nutrition guidelines, and their preferences for how to eat more healthily. This is an essential consideration for the development of tailored interventions aimed to help people adopt changes in their dietary behavior.

**Methods:**

Seventy-eight participants filled in an online questionnaire containing both open-ended and closed-ended questions. The qualitative data was analyzed using content analysis resulting in a classification scheme. Two students then identified to which category each part of a participant’s answer belonged.

**Results:**

For both the perception of a healthy diet and how to eat healthily, four major categories and a residual category were identified: dietary patterns, food processing, food products, content/nutrients, and non-food. These major categories consisted of several categories. The results showed that how people perceived a healthy diet was mostly represented at the level of food product (vegetables and fruit) and the content/nutrient level (carbohydrates), whereas how they would like to eat healthily was mostly represented at the level of food processing (preparation), food product (vegetables), and dietary patterns (amount).

**Conclusions:**

Our findings are mostly in line with how the Dutch dietary guidelines are communicated (“product level”). However, consumers primarily mention single aspects instead of naming the guidelines as a whole. Health policymakers can use this insight in future communications regarding the guidelines to the general public. A challenge for future (eHealth) diet interventions is how to implement and tailor dietary information that optimally connects with the perceptions of the target population.

**Supplementary Information:**

The online version contains supplementary material available at 10.1186/s40814-020-00735-6.

## Key messages regarding feasibility

- What uncertainties existed regarding the feasibility? Based on our findings, we have identified eating behaviors that people perceive as part of a healthy diet and ways to improve their dietary patterns. One of the challenges for self-determined (eHealth) interventions for dietary behavior is how to implement and tailor this information to optimally connect to the target population.
- What are the key feasibility findings? Our key finding is more insight into how people define a healthy diet and their preferred ways of eating more healthily. These perceptions slightly differ from the scientifically quantified definition and understanding of what healthy eating entails. The newest insight was that participants mentioned specific elements of the Dutch dietary guidelines instead of naming them as a whole, and they generally provided no portion sizes. These insights are incorporated into the intervention.
- What are the key implications of the feasibility findings for the design of the main study? The key implications of the feasibility findings was that this pilot study derived information to tailor our intervention to the target population’s needs so that they can more easily relate to the intervention and to operationalize our main behavioral targets by linking them to the needs, perception, and language of the target population. Furthermore, some brief information about the Dutch dietary guidelines is included in the intervention.

## Background

Unhealthy dietary patterns are highly prevalent in Western countries, and they have been associated with depression, hypertension, heart disease, cancer, type 2 diabetes, and obesity [1]. Therefore, the promotion of a healthy dietary intake is important for public health. The small effect sizes, limited sustainability of effects, and high dropout rates usually found for existing online diet interventions show a need for improvement in this field [2]. One way to make dietary interventions effective is by making them individualized or tailored [3]. The purpose of this study was to assess how people define a healthy diet and what their preferred ways are of eating healthily. The insight provided by this study can help to develop better interventions optimally tailored to the individuals in a target population.

National and international dietary guidelines provide advice and principles on meeting healthy nutritional patterns to maintain health and reduce disease risk. However, most adults in the Western world do not comply with these recommendations [4, 5]. The Netherlands Nutrition Centre has translated the guidelines provided by the Health Council of the Netherlands into a practical visual tool, called the “Wheel of Five,” to give examples of healthy dietary patterns to the general public [6, 7]. This wheel is divided into four food groups and one beverage group, as described in Table 1. The Wheel of Five has mainly been formulated in terms of edible products, making it easier for consumers to make healthier choices.

**Table 1:** A short overview of the Dutch dietary guidelines for adults

Food group	Recommended daily amounts^a^
1. Vegetables and fruit	250 g of vegetables 200 g of fruit
2. Spreading and cooking fats^a^	35–65 g
3. Dairy, nuts, fish, legumes, meat, and eggs^a^	1 portion of fish^b^/meat/legumes/eggs 15–25 g of unsalted nuts 2–4 portions of dairy products
4. Bread, grain/cereal products, and potatoes^a^	3–8 slices of whole-grain bread 3–6 serving spoons of whole-grain products or 3–6 potatoes
5. Drinks	1.5–2 l of drinks without sugar

More detail regarding the Dutch dietary guidelines can be found elsewhere [6, 7]

^a^The numbers are dependent on age and sex

^b^It is advised to consume fish once a week

Researchers and nutritionists have developed a scientifically quantified definition and a clear understanding of what healthy eating entails. This definition has been operationalized as guidelines for which portion sizes have been provided. However, it is doubtful whether the general population shares this understanding of a healthy diet, as they are continually being confronted with a wide variety of nutritional information consisting of both proven and unproven health claims. Though people intend to eat more healthily, many do not succeed [8, 9]. This may not be very surprising since eating more healthily is typically seen as a set of challenging and complex behaviors [10]. For this purpose, it is essential to examine what people perceive as a healthy diet, whether this is in line with the dietary recommendations, and how people prefer to eat healthily. This knowledge may inform the design of interventions aimed at promoting healthy eating. Research on the current perceptions of a healthy diet in the population, specifically Dutch adults, is limited.

Until now, several studies have addressed the perceptions of a healthy diet, but most of these studies were conducted over 20 years ago. One large pan-European study grouped participants’ perceptions into eight major categories that captured healthy eating. These categories were less fat, more fruit and vegetables, balance and variety, fresh/natural foods, less (red) meat, nutrient approach, fewer staples/fibers, and less sugar [11]. Another study found that more fish and more lean meat were also part of the healthy eating definition in a large Spanish sample [12]. A more recent study found that lower-educated Dutch, Turkish, and Moroccan adults living in the Netherlands with a lower vocational level or less considered mainly fruit and vegetables as part of a healthy diet [13]. Thus, fruit, vegetables, avoiding or limiting meat, balance/variety/moderation, and less fat seem to constitute a healthy diet according to many consumers; these elements were also identified in a review by Paquette [14]. These elements are also consistent with parts of the dietary guidelines, but the descriptions are less detailed than the actual guidelines. Scientific studies and new insights have advanced considerably in the last decades, and nutritional guidelines have evolved to keep pace with the latest findings and with the changing patterns in food consumption [15]. It is questionable if the average consumer’s understanding of healthy eating corresponds to these professional insights and dietary guidelines. So far, research on the current perceptions of a healthy diet in the population remains scarce.

This pilot study aimed to identify what Dutch adults perceive as a healthy diet and examine what they perceive as the preferred ways of eating more healthily. A better understanding of what Dutch consumers perceive as a healthy diet, how this relates to the current Dutch dietary guidelines, and their preferences for eating more healthily is important for the development of tailored interventions aimed at helping people adopt changes in their dietary behavior. This insight can be used to make interventions more effective in the following ways. Firstly, the target behaviors of the intervention, such as fruit intake, can be chosen for evaluation purposes. Secondly, information regarding the dietary guidelines can be tailored to the needs of the user. Thirdly, the language that the consumers use to describe their perceptions can be implemented in the intervention so that they can more easily relate to the intervention. On a higher level, this study provides input for health policymakers, namely, to bring to their attention what information about specific food groups is missing or what misperceptions are present that can be tackled in future communications regarding the guidelines.

## Methods

### Design, participants, and procedure

This study primarily used a qualitative design. We aimed to recruit at least 50 Dutch-speaking adults who were at least 18 years old using a short advertisement on social media (Facebook) between July and August 2017 [16]. This advertisement contained a colorful image of healthy food and a message asking for people’s opinion on healthy food and stating that they could win a gift voucher worth 20 euros. People who were interested in participating could click on the link to the study website containing more information about the study. Subsequently, they could enroll in the study by filling in an online consent form. Participants were then able to proceed to the online questionnaire, which took a maximum of 15 min to complete. As an incentive for participation, respondents who completed the questionnaire were entered into a raffle for a gift voucher. The study was approved by the ethical committee of the Open University of the Netherlands (reference number: U2017/04575/FRO).

### Instrument

A short online questionnaire was created to assess participants’ perceptions of a healthy diet. To assess what people consider as healthy eating and how they would prefer to eat healthily, two open-ended questions were asked. Questions that were asked were “what do you perceive as healthy eating in terms of products, amounts, preparation, etc.?” followed by “how would you prefer to eat healthily?”. Participants could answer these questions in an open-text field with no word count restrictions. In addition to these open-ended questions, participants had to select up to four dietary practices among 24 predefined possibilities about how they would like to eat healthily. These dietary practices were based on dietary guidelines, such as eating more vegetables, as well as marketing hypes, for example, eating superfoods. Participants also reported their age, gender, the highest level of education completed, weight (optional), and height. Furthermore, participants also rated how important healthy food was for them on a Likert scale ranging from 1 (not important at all) to 10 (extremely important).

### Data analysis

The responses to the open-ended questions were analyzed using conventional content analysis, aiming to create categories of healthy eating [17]. A coding tree was developed based directly on the text data in the following way.

#### Data preparation

First, all data were inspected by the lead author and a research assistant for an overall understanding and identification of categories, which were also based on previous literature [6]. Furthermore, obvious typing errors were corrected, e.g., “began” was corrected to vegan.

#### Development of coding scheme

The answers were read word by word and were split into meaningful segments (“codes”) that appeared to capture key thoughts or concepts. For example, the response “healthy food consists of a good balance between different nutrients (fat/carbohydrates/proteins) and contains sufficient vitamins and minerals (participant 63)” was segmented into “healthy food consists of a good balance,” “different nutrients,” “fat,” “carbohydrates,” “proteins,” “(sufficient) vitamins,” and “minerals.” Colored tags were then used to organize the responses into categories, such as nutrients or carbohydrates. Upon further inspection of the data, concept categories were refined to optimally fit the data. These preliminary categories were discussed with Lilian Lechner, Catherine A. W. Bolman, and Anke Oenema, and an initial categorization scheme was developed.

#### Procedure of coding

Two graduate psychology students were directed to also split the answers of the respondents into meaningful segments. They then used the initial coding scheme to identify to which category each segment belonged. The initial inter-rater reliability was 93.4% for how participants defined a healthy diet and 91.9% for how to eat more healthily. Discrepancies were discussed with JC, and revisions to the categorizations were made until full consensus was achieved. After this categorization process had been completed, further higher-order categories (dietary patterns, food processing, food product, content/nutrient, and non-food) were identified to construct a broader definition of healthy eating and preferred ways of eating healthily. We recorded the frequency and percentage of each (higher) category. Also, for the closed-ended question in which participants selected several options among a predefined list of ways of eating more healthily, we calculated how many participants (%) mentioned each unique category. The results of the questionnaire were not returned to the participants for feedback. Microsoft Excel was used for the qualitative analyses. Furthermore, we filled in the Standards for Reporting Qualitative Research (SRQR) to report important aspects of the study (Supplementary File) [18].

## Results

### Descriptive statistics

In total, 78 Dutch adults (51 females, 65%) enrolled in this study. Age varied between 18 and 68 years (*M*_age_ = 38.4 years, SD_age_ = 15.5). More than half of the respondents (*n* = 43, 55%) had completed higher-level professional education, university or postgraduate studies, while 20 respondents (26%) had graduated from high school (with the Dutch HAVO or VWO qualifications) or had a degree in middle professional education. Fourteen respondents (18%) had no education beyond primary school, had graduated from lower-level secondary education, or had completed lower professional education. One respondent replied with “other education.” The mean BMI of the sample was 23.3 ± 4.1. Of this sample, four respondents (5%) were classified as underweight, 51 (65%) as having a normal weight, 15 (19%) as non-obese overweight, and 5 (6%) as obese. Three respondents did not fill in their weight (4%). The mean importance of a healthy diet for this sample was 8.3 ± 1.4 (scale range 1–10).

### What is healthy eating?

Four major categories and a residual category were constructed made up of phrases defining healthy eating in this sample. The largest category “food products” consisted of drinks, fish, fruit, grain products, meat, nuts, specific food products (i.e., products that could not be grouped in the other food product categories), and vegetables. The second largest “content/nutrients” contained the following categories: calories, carbohydrates, fat, fibers, nutrients, protein, salt, and vitamins/minerals. This major category was followed by “food processing” that consists of two categories: preparation and organic/sustainable foods. The following major category was “dietary patterns” consisting of four categories: type of eater, amount, balanced, and guidelines. The smallest major category was “non-food” containing “others” or segments that could not be classified into the other categories, hype/claims, and lifestyle. See Table 2 for an overview of all the (major) categories that were identified. The frequencies and percentages, calculated based on all meaningful segments mentioned by all participants, are presented in the fourth column. How often each individual category was mentioned by participants is displayed in the fifth column. From all segments given, most belonged to the following categories: vegetables, organic/sustainable, fruit, preparation, carbohydrates, fat, amount, and balanced. The least frequent categories were hype/claims (“non-food”), calories (“content/nutrient”), grain products (“food product”), type of eater, and guidelines (“dietary patterns”).

**Table 2:** Overview of the categories on what is healthy eating

Major categories (*n*, %)	Categories	Examples	Frequencies (answers; %)^a^	Frequencies (pp; %)^b^
Dietary patterns (52, 12.5%)	Balanced	Varied; balanced	22 (5.3)	19 (24.4)
	Amount	Right amount; moderated	21 (5.1)	19 (24.4)
	Guidelines	Wheel of Five	5 (1.2)	5 (6.4)
	Type of eater	Vegetarian; vegan	4 (1.0)	4 (5.1)
Food processing (79, 19.0%)	Organic/sustainable	Unprocessed; biological	42 (10.1)	31 (39.7)
	Preparation of food	Healthy preparation; fresh; no pre-prepared food	37 (8.9)	27 (34.6)
Food product (148, 35.7%)	Vegetables	Vegetables; legumes	46 (11.1)	42 (53.9)
	Fruit	Fruit	39 (9.4)	39 (50.0)
	Meat	Not too much meat; chicken	15 (3.6)	14 (18.0)
	Drinks	A lot of water; herb tea	13 (3.1)	11 (14.1)
	Nuts	Nuts; seeds	12 (2.9)	8 (10.3)
	(Specific) food products	Eggs; dairy	11 (2.7)	10 (12.8)
	Fish	Fish	7 (1.7)	7 (9.0)
	Grain products	Bread; grains	5 (1.2)	5 (6.4)
Content/nutrient (116, 28.0%)	Carbohydrates	Fast carbs; no sugar	32 (7.7)	18 (35.9)
	Fat	Good fats; low in fat	27 (6.5)	25 (32.1)
	Fibers	Rich of fibers	15 (3.6)	15 (19.2)
	Salt	Little salt	14 (3.4)	14 (18.0)
	Vitamins/minerals	Vitamins	10 (2.4)	10 (12.8)
	Nutrient	Nutrients; nutritional value	7 (1.7)	7 (9.0)
	Protein	Proteins	7 (1.7)	7 (9.0)
	Calories	Few calories	4 (1.0)	4 (5.1)
Non-food (20, 4.8%)	Lifestyle	Exercising	10 (2.4)	10 (12.8)
	Others	Development in the food industry	8 (1.9)	8 (10.3)
	Hype/claims	Do not follow hypes	2 (0.5)	2 (2.6)

Owing to rounding, percentages do not always add up to 100%

^a^In this column, it can be found how often this category was mentioned in all answers (*n* = 415)

^b^In this column, it can be found by how many participants a particular category was mentioned (*n* = 78)

### How to eat healthily?

The same four major categories and a residual category could be applied to the categories of how to eat healthily. Here, the largest category was “food processing” that consists of two categories: preparation and organic foods. The second largest major category was “food products” consisting of dairy, drinks, fish, fruit, grain products, meat, nuts, (healthy) products, snacks, and vegetables. This major category was followed by “dietary patterns” consisting of five categories: type of eater, amount, balanced, eating pattern, and guidelines. The following major category was “content/nutrients” that contained the following categories: calories, carbs, fat, nutrients, and protein. The smallest major category was “non-food” containing segments that could not be classified in the other categories, environment, information, and shopping. See Table 3 for an overview of all (major) categories that were identified for how to eat more healthily, presented with the frequencies and percentages, calculated based on all phrases (*N* = 197) and how often each individual category was mentioned by participants. We found that segments that belonged to the preparation category were mentioned most often, i.e., about 40% of this sample mentioned concepts belonging to this category. From all segments given, most belonged to the following categories: preparation, balanced, vegetables, and organic. The least frequent categories were nuts, dairy (“food product”), protein (“content/nutrients”), and environment (“non-food”).

**Table 3:** Overview of the categories on how to eat more healthily

Major categories (*n*, %)	Categories	Examples	Frequencies (answers; %)^a^	Frequencies (pp; %)^b^
Dietary patterns (49, 24.9%)	Balanced	Varied	21 (10.7)	21 (26.9)
	Amount	Moderated; small portions	12 (6.1)	12 (15.4)
	Type of diet/eater	Vegan or vegetarian	7 (3.6)	7 (9.0)
	Eating pattern	Six fixed eating occasions	7 (3.6)	7 (9.0)
	Guidelines	Advice; Wheel of Five	2 (1.0)	2 (2.6)
Food processing (59, 29.9%)	Preparation of food	Stir fry; home prepared	43 (21.8)	31 (39.7)
	Organic	Natural products	16 (8.1)	14 (18.0)
Food product (54, 27.4%)	Vegetables	A lot of vegetables	16 (8.1)	16 (20.5)
	(Healthy) products	Healthy products	9 (4.6)	9 (11.5)
	Fruit	Fruit	7 (3.6)	7 (9.0)
	Snacks	No snacks	7 (3.6)	7 (9.0)
	Grain products	Less bread; multigrain products	5 (2.5)	5 (6.4)
	Meat	No or less meat	4 (2.0)	4 (5.1)
	Drinks	Smoothie	2 (1.0)	2 (2.6)
	Fish	Fish	2 (1.0)	2 (2.6)
	Nuts	Nuts	1 (0.5)	1 (1.3)
	Dairy	Dairy	1 (0.5)	1 (1.3)
Content/nutrient (19, 9.6%)	Carbs	Decrease sugar intake	9 (4.6)	9 (11.5)
	Fat	Less fat; healthy fat	5 (2.5)	5 (6.4)
	Nutrients	Nutritional values	2 (1.0)	2 (2.6)
	Calories	Calories	2 (1.0)	2 (2.6)
	Protein	Rich of proteins	1 (0.5)	1 (1.3)
Non-food (16, 8.1%)	Shopping	Groceries shopping; buying	8 (4.1)	8 (10.3)
	Others	Keep it simple	5 (2.5)	5 (6.4)
	Information	Gain knowledge	2 (1.0)	2 (2.6)
	Environment	At the table	1 (0.5)	1 (1.3)

Owing to rounding, percentages do not always add up to 100%

^a^In this column, it can be found how often this category was mentioned in all answers (*n* = 197)

^b^In this column, it can be found by how many participants a particular category was mentioned (*n* = 78)

### Preferred ways of eating healthier

**Fig. 1:**
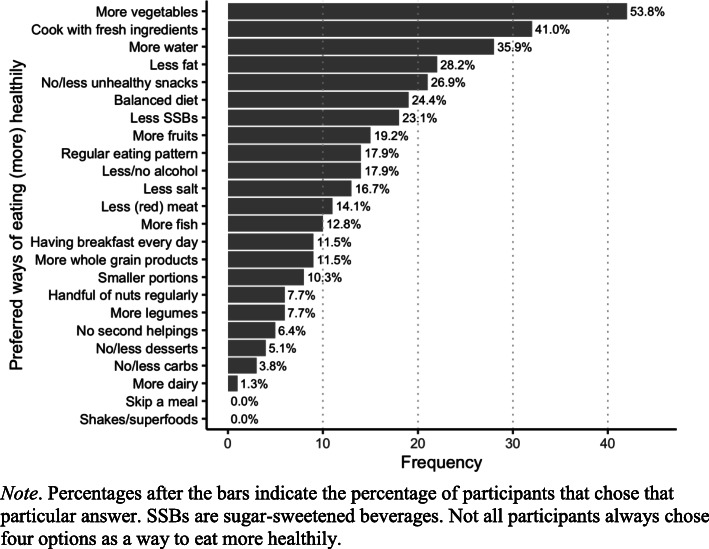
Overview of the preferred ways to practice a healthy diet based on selection among predefined categories

Participants also had to select four options that represented their preferred way of eating healthily among predefined categories. These results are presented in Fig. 1. We found that eating more vegetables was chosen by more than half of the participants as a preferred way to eat more healthily (*n* = 42, 54%). Furthermore, cooking with fresh ingredients (*n* = 32), drinking more water (*n* = 28), and limiting fat intake (*n* = 22) were other preferred ways to practice a healthy diet.

## Discussion

This qualitative pilot study examined the perception of a healthy diet and the preferred ways of eating more healthily of Dutch adults. The results showed that, to a large extent, similar categories were identified for both the perception of a healthy diet (*N* = 25) and the preferred ways people would eat more healthily (*N* = 26). These categories correspond to constructs that have been named in other Western countries, such as fat, fruit/vegetables, balance and variety, fresh/natural foods, meat, nutrients, fibers, sugar, and fish [11, 12, 14]. As can be seen from the total number of categories, our study used a more detailed approach to group data than other studies, resulting in more categories [11, 12, 14]. Looking at all the identified categories in more detail, it was found that various categories were related to each other in content. These categories could be grouped into four higher-level categories and a more residual category: dietary pattern, food processing, food product, nutrients, and non-food.

Our study illustrates that how our participants define a healthy diet does not necessarily correspond to how they would like to eat more healthily. The findings regarding the perception of a healthy diet demonstrated that most well-represented phrases focused on the food product level, such as vegetables and fruit, and the content/nutrient level, such as carbohydrates. On the contrary, the preferred ways of eating healthier were mostly described at the level of food processing, food product, such as vegetables, and in terms of dietary patterns, e.g., the portion size. This is in line with previous studies in which many participants named fruit and vegetables as part of a healthy diet [11–13].

This study also showed that people are generally inclined to think about food products instead of nutrients. People may not necessarily think about what makes particular food healthy. These ideas correspond to how The Netherlands Nutrition Centre has communicated the Dutch guidelines for a healthy diet: almost wholly in terms of food products and dietary patterns, such as amounts. This description relates better to recent scientific developments and food choices as perceived by the general public [6]. Additionally, most answers within the content/nutrient category contained statements regarding fat/carbohydrates, showing that the lowering of the intake of foods that are high in fat and sugar is perceived as important for eating more healthily.

Only five participants mentioned the dietary guidelines or the Wheel of Five explicitly. A previous study has identified that about 92% of Dutch people reported having heard of the Wheel of Five [7]. Although many participants may have heard of the Wheel of Five, this representation of the Dutch dietary guidelines does not entirely seem to represent their ideas of a healthy diet. They only seem to think about some aspects of the guidelines rather than the dietary guidelines as a whole, i.e., naming the Wheel of Five explicitly or naming all elements of a healthy diet that constitute the Dutch dietary guidelines. This was the case even when the participants’ full combined answers were considered.

Another remarkable finding was the frequent occurrence of how the food was prepared and whether it was organic. The latter result could be related to the misperception that organic food is always more healthy than conventional foods. Several reviews found no evidence of a difference in nutrient quality between organic and conventional foods; however, consuming organic foods may lower exposure to pesticide residues and antibiotic-resistant bacteria [19, 20]. On the other hand, it has been found that organic food has been linked to a lower risk of overweight/obesity [21]. This may be due to an overall healthier lifestyle of consumers who regularly buy organic foods; they are more likely to buy more vegetables, fruit, whole grain products, and less meat, and they are also likely to be more physically active and less likely to smoke [21, 22].

Furthermore, when the participants selected several options among an extensive list of ways of eating more healthily, a similar pattern could be seen. More than half of the study participants preferred eating vegetables as a way to eat more healthily. They also selected cooking with fresh ingredients, drinking more water, and limiting fat intake quite frequently. However, phrases belonging to these categories were mentioned less often in the open-ended questions. This might be because people recognize these options as healthy, but they do not necessarily come to mind in the open-ended questions. Even though the study’s focus was on food, drinks, such as water or sugar-free drinks, are essential for a healthy diet according to the dietary guidelines. Participants in our study also did not seem to think about this when asked about their definition of a healthy diet.

Some limitations of this study should be noted. Since food intake was not assessed in this sample, we cannot link eating behavior to perceptions of a healthy diet. More research is needed on how these perceptions affect actual food choices. A second limitation was the use of self-selection sampling. People could take part of their own accord. This may have resulted in a highly educated sample and maybe, therefore, an overrepresentation of people with a healthy weight and who perceived a healthy diet as quite important. This may limit generalizability to the general population. However, participants in the dietary intervention for which this pilot study has been conducted can also take part in their own accord. Therefore, it is expected that these results do represent the targeted intervention population to some extent. Future studies could examine whether the categories we have identified for the definition of a healthy diet and the preferred ways of eating more healthily still apply in a larger and more diverse sample, especially among people with overweight and low education levels. This population segment eats less healthily and may, as a consequence, have different perceptions of what a healthy diet entails or vice versa [23]. We found some first indications that women gave more vegetable-related, fruit-related, and meat-related answers in their definition of a healthy diet. On the other hand, younger participants provided more type-of-eater-related statements, and men gave more portion size-related answers for their preferred ways of eating more healthily. However, these findings were no longer significant after the correction for multiple testing. Although these findings can mainly be considered as tentative, they do provide input for the direction of follow-up research. These differences in perceptions between subgroups may also provide indications for other emphases in interventions.

This study has important theoretical and practical implications. This study not only provided a more up-to-date view on how this group of Dutch adults experience and describe components of a healthy diet, but also determined the preferred ways of eating more healthily, as expressed in their own words. It demonstrated that information about a healthy diet does not fully correspond to people’s preferences on how to eat more healthily. This finding has important implications for developing dietary interventions. By giving people the option to choose for themselves what behavior to work on within an intervention, their behavioral change could be promoted. This change could be enhanced even further when consumers’ terminology is used. In this sense, a form of relatedness is created that promotes more autonomous types of motivation to eat healthily, and in turn, stimulates behavioral change [24]. Furthermore, these findings demonstrate that it is important to provide a full overview of the guidelines within an intervention, as most participants in this pilot study did not mention all aspects of the guidelines. However, as our closed-ended question regarding the preferred ways to eat healthily showed, some of these aspects are still selected but not frequently mentioned when participants have to come up with their own ideas, which is the case for water consumption. Participants can then make an informed choice on what behavior they would like to work on. Another implication might be that some aspects of the guidelines may need extra attention as they can be recognized but not recalled. For example, water consumption is frequently chosen in the closed-ended question to engage in a healthier diet, but this has not often been named in the open-ended questions. Health policymakers can strengthen active recall by bringing the consumption of certain healthy food products, such as water, to the general population’s attention.

### Conclusion

In conclusion, the participants’ definition of a healthy diet does not fully correspond to how they would like to eat more healthily. Nevertheless, the perceptions of healthy eating are influenced by some dietary principles, e.g., the consumption of vegetables and fruit. However, participants do not mention quantities/sizes and only name some elements of the guidelines. Other elements that also seem important to people’s perceptions of healthy eating, such as how the food is prepared, whether it is organic, and limiting sugar and fat intake, are not explicitly named in current dietary guidelines. Our results also suggest the need to improve nutritional education or strengthen recall about certain healthy food products, such as water consumption. For future dietary guideline updates and especially the communication about them to the public, it is essential that these guidelines better match the perceptions of the population (or vice versa). One of the challenges for self-determined (eHealth) interventions for dietary behavior is how to implement and tailor this information to optimally connect to the target population, as everyone has their own perceptions of a healthy diet and preferred ways of eating more healthily.

## Supplementary Information

**Additional file 1:** Standards for Reporting Qualitative Research (SRQR)*

## Data Availability

The study data and materials of this study are available upon request.
